# Mapping personal recovery in schizophrenia spectrum disorders: an exploratory machine learning study of self-reported stage classifications

**DOI:** 10.3389/fpubh.2026.1822191

**Published:** 2026-06-15

**Authors:** Anaid Pérez-Ramos, Julien Plasse, Isabelle Chéreau-Boudet, Benjamin Gouache, Emilie Legros-Lafarge, Nathalie Guillard-Bouhet, Nicolas Franck, Guillaume Barbalat

**Affiliations:** 1 Centre for Biomedical Research in Mental Health (CIBERSAM), ISCI-III, Madrid, Spain; 2 Barcelona Clinic Schizophrenia Unit (BCSU), Neuroscience Institute, Hospital Clinic of Barcelona, Barcelona, Spain; 3 Institut d'Investigacions Biomèdiques August Pi i Sunyer (IDIBAPS), Barcelona, Spain; 4 University of Cadiz, Cadiz, Spain; 5 Centre Ressource de Réhabilitation Psychosociale et de Remédiation Cognitive (CRR), Hôpital Le Vinatier, Centre National de la Recherche Scientifique (CNRS UMR 5229) et Université Lyon 1, Lyon, France; 6 Centre Référent Conjoint de Réhabilitation (CRCR), Centre Hospitalier Universitaire de Clermont-Ferrand, Clermont-Ferrand, France; 7 Centre Référent de Réhabilitation Psychosociale et de Remédiation Cognitive (C3R), Centre Hospitalier Alpes Isère, Grenoble, France; 8 Centre Référent de Réhabilitation Psychosociale de Limoges (C2RL), Limoges, France; 9 Centre de REhabilitation d'Activités Thérapeutiques Intersectoriel de la Vienne (CREATIV), Centre Hospitalier Laborit, Poitiers, France

**Keywords:** personal recovery, random forest, REHABase, schizophrenia, SHAP values, subjective recovery

## Abstract

Recovery in schizophrenia is a complex and multidimensional process involving functional, clinical, and psychological dimensions. This study aims to use machine learning (ML) to explore multivariate associations with classifications of personal recovery assessed with the Stages of Recovery Instrument (STORI). We analyzed cross-sectional data from 1,361 individuals with schizophrenia-spectrum disorders enrolled in the French REHABase cohort, a national psychosocial rehabilitation network. Random forest models were developed using internal cross-validation on a training set and subsequently evaluated on an independent test set (70/30 split). Model performance was high in the training data (AUC = 0.997; accuracy = 94.3%) and more modest in the test set (AUC = 0.724; accuracy = 49.8%). SHAP analyses (explainable ML) were used to describe stage-associated recovery profiles based on multivariate patterns across sociodemographic, clinical, and psychological variables. Overall, the modest performance observed in the independent test set underscores the complexity of personal recovery and is consistent with partial conceptual overlap across STORI stage classifications. The Moratorium stage was characterized by higher negative self-esteem and internalized stigma, alongside lower wellbeing and positive self-esteem. Awareness was associated with mental wellbeing and negative self-esteem, with additional contributions from positive self-esteem and relational satisfaction. The Preparation stage showed less distinct profiles, with autonomy emerging as a relevant feature. Rebuilding was associated with intermediate-to-high levels of wellbeing and resilience. The Growth stage was characterized by high wellbeing, positive self-esteem, and relational satisfaction. These findings provide an integrated description of stage-associated recovery profiles and illustrate how explainable ML can be used to explore the multidimensional organization of personal recovery.

## 1 Introduction

Epidemiological data from recent years reflect an increase in the prevalence and economic burden of mental health problems. In 2019, the global prevalence of mental disorders remained high, with 1 in every 8 people worldwide living with a mental disorder (1). In particular, schizophrenia spectrum disorders (SSD) represent a major challenge in mental health care. Schizophrenia is a chronic illness with a highly heterogeneous course that has traditionally been associated with a poor prognosis (2). However, a substantial proportion of individuals with schizophrenia achieve symptom remission and meaningful psychosocial functioning (3).

In this context, the concept of recovery has gained increasing recognition in the field of mental health. Recovery-oriented approaches have been increasingly integrated into international mental health policies (4, 5), emphasizing their potential to enhance patient outcomes while reducing long-term healthcare costs. Recovery is understood as a multidimensional and individualized process that encompasses both subjective aspects, such as wellbeing, quality of life, and self-esteem, and objective aspects, including social functionality and independence in daily activities (6). Different conceptualizations of recovery have been proposed. On the one hand, clinical recovery is defined as symptom remission and restoration of functioning, maintained for at least 6 months (7). On the other hand, functional recovery refers to the ability of a person with stabilized symptoms to live independently for an extended period, maintain meaningful social relationships, and perform academic or work roles without relapse (8). However, these definitions do not necessarily capture how the person themselves experiences their recovery process.

Subjective or personal recovery refers to the individual's own perception of their recovery journey, emphasizing empowerment, hope, identity, meaning in life, and overcoming stigma (9), and is widely understood as a subjective, individualized, and non-linear process rather than a fixed or uniform developmental sequence (10–12). Previous research has explored factors that significantly influence personal recovery, such as self-stigmatization, hopelessness, and self-esteem (13, 14). Likewise, numerous studies have demonstrated that an individual may meet clinical remission criteria and yet not feel recovered from a personal perspective (6, 15). This dissociation highlights that clinical and functional definitions of recovery provide only a partial view of the patient's actual state and may overlook key processes related to wellbeing and long-term recovery. Few instruments have been specifically designed to quantitatively assess the recovery process and the stages of recovery in mental health (16). The Stages of Recovery Instrument (STORI) (17) offers a theoretical framework to understand the personal recovery process, based on a five-stage model. These stages (moratorium, awareness, preparation, rebuilding, and growth) have traditionally been described as reflecting a conceptual progression from a state of hopelessness, through the development of insight and preparation for change, to active rebuilding of life and personal growth. However, the conceptualization of these stages as strictly progressive and ordinal has been increasingly questioned. Psychometric studies have highlighted substantial conceptual overlap between stages, suggesting that the model may not accurately capture the five distinct stages proposed originally (18). These findings challenge the assumption of a uniform and sequential progression, supporting instead a more dynamic and non-linear understanding of personal recovery. Despite these conceptual debates, previous studies have demonstrated that individuals classified in higher STORI stages of recovery have been associated with lower stigma, greater insight, better coping strategies, and enhanced quality of life (19), supporting its construct validity and clinical relevance.

Given the complex and subjective nature of personal recovery, it is pertinent to explore analytical approaches capable of simultaneously integrating a wide range of clinical, sociodemographic, and psychological variables. Machine learning (ML), a branch of artificial intelligence, could offer promising methods to uncover complex patterns in large datasets. Unlike traditional statistical methods, which often rely on predefined assumptions and linear relationships, ML algorithms can model nonlinear interactions and handle high-dimensional data. A recent study has demonstrated the utility of ML in schizophrenia, including the prediction of diagnosis, symptom trajectories, and complications such as insomnia, depression, suicide risk, and cognitive decline (20). Yet, its potential to explore the complexity and multidimensionality of personal recovery remains largely unexplored, as no previous studies to our knowledge have applied ML to examine how different variables are differentially associated with stages of personal recovery. The aim of this study was to examine multivariate patterns associated with STORI-defined recovery stages using machine learning. Specifically, we sought to characterize how sociodemographic, clinical, functional, and psychological variables are differentially associated with recovery stage classifications and to identify stage-specific recovery profiles.

## 2 Material and methods

### 2.1 Study design and data source

This study used a cross-sectional, retrospective design based on baseline data derived from an ongoing longitudinal cohort (REHABase). We included 1,361 participants, from 33 centers of a French psychosocial rehabilitation (PSR) network (21). The cohort includes patients with serious mental illness referred to the centers by public mental health services, private psychiatrists, and general practitioners or self-referred. Patients included in the cohort subsequently benefit from a personalized rehabilitation care plan that can last from a few months to a year. Patients are included if they exhibit (i) significant functional impairment resulting from their mental illness, which markedly disrupts one or more major life activities (22); (ii) minimal clinical stability; and (iii) clear commitment to participate in PSR programs. All assessments included in the present study correspond to baseline evaluations conducted at entry into the PSR program, reflecting participants' clinical and psychosocial status at a relatively stabilized phase of the illness.

Our analysis included patients recruited between 2016 and 2024 with a DSM-5 (Diagnostic and Statistical Manual of Mental Disorders, 5th edition) diagnosis of SSD based on a clinical interview conducted by a psychiatrist (23). Diagnostic assessments were conducted within specialized psychosocial rehabilitation centers operating under standardized clinical protocols, ensuring diagnostic consistency across sites. All clinical information was collected within specialized psychosocial rehabilitation centers following standardized assessment procedures routinely implemented in the REHABase network.

The database received the necessary authorizations in compliance with French regulations, including approval from the French National Advisory Committee for the Treatment of Information in Health Research (16.060bis) and the French National Computing and Freedom Committee (DR-2017-268). All participants provided written informed consent prior to inclusion in the cohort, in accordance with national ethical standards and the Declaration of Helsinki.

### 2.2 Outcome variable

The STORI was used as the outcome measure, as it was the personal recovery instrument available in the REHABase cohort at the time of data collection. It is important to note that while recovery frameworks such as CHIME have provided influential multidimensional conceptual models, they do not correspond to a single, standardized measurement instrument. In contrast, the STORI enables an operationalized assessment of recovery experiences at the individual level, allowing participants to be classified into recovery profiles. Furthermore, systematic reviews have shown that the STORI demonstrates a relatively broad range of psychometric properties compared to other available recovery instruments, while capturing key experiential aspects of personal recovery. In particular, the systematic review by Shanks et al. (24) identified the STORI as one of the measures reporting the highest number of evaluated psychometric properties among recovery instruments, and a subsequent review highlighted both the heterogeneity of recovery measures and the absence of a single optimal instrument (25).

The STORI (17) measures the personal aspect of recovery. It is a 50-item self-report questionnaire, where each group of 10 items corresponds to one of the five stages of recovery. Items are rated from 0 “not at all true now,” to 5 “completely true now,” producing a score for each stage ranging from 0 to 50. The instrument has demonstrated adequate psychometric properties (26, 27). Five stages of recovery are:

1. Moratorium: A time of withdrawal, denial of illness identity with a profound sense of loss and hopelessness.
2. Awareness: Realization that all is not lost, and that a fulfilling life is possible.
3. Preparation: Taking stock of strengths and weaknesses regarding recovery, and starting to work on developing recovery skills.
4. Rebuilding: Stage of active work toward a positive identity, setting meaningful goals, and taking control of one's life.
5. Growth: Living a full and meaningful life, characterized by self-management of illness, resilience, and a positive sense of self.

The stage of recovery is determined by the highest average score a participant obtains across the five-stage subscales. In cases where two subscales have the same score, the participant is assigned to the higher stage.

Although the STORI was originally conceptualized as a progressive sequence of recovery stages, several empirical observations challenge such a strict interpretation (18, 28). In our sample, strong positive correlations were observed not only between adjacent stages but also between non-adjacent ones, indicating considerable overlap between recovery dimensions (see Supplementary Table 1). Therefore, to avoid imposing assumptions of ordinality or unidirectional progression, in this study the STORI stage was conceptualized as a categorical outcome variable. This approach makes it possible to identify differences between recovery profiles while taking into account the conceptual overlap inherent in the STORI.

For analytical purposes, the five STORI stages were coded as follows: X1 = Moratorium, X2 = Awareness, X3 = Preparation, X4 = Rebuilding, and X5 = Growth.

### 2.3 Recovery-related variables

A total of 38 potential recovery-related variables were used in the analysis. These include sociodemographic, clinical, and psychological variables. Clinical variables and sociodemographic data were collected by clinicians as part of routine baseline assessments. All self-report scales were administered in French, using validated French versions when available. Participants completed the questionnaires autonomously, with assistance from trained clinicians if clarification was required.

Sociodemographic variables included age, sex, education level, family situation, parental status, housing situation, employment status, social marginalization, and medico-legal history. Clinical variables comprised comorbid diagnoses, number of prescribed medications, type of addiction, origin of initial referral, duration of illness, number of psychiatric admissions, duration of hospitalization, suicide attempts, medication adherence, global functioning, and illness severity scores. Illness severity was assessed using the Clinical Global Impression (CGI) (29), global functioning using the Global Assessment of Functioning (GAF) (30), and medication adherence using the Medication Adherence Rating Scale (MARS) (31).

Psychological variables included self-esteem, insight, internalized stigma, quality of life, and wellbeing. Self-esteem was assessed through two subscales of the Self-Esteem Rating Scale (SERS), reflecting positive and negative self-esteem, respectively. In addition, we also included the self-esteem subscale from the S-QoL 18, reflecting a more future-oriented aspect of the construct, such as optimism and confidence in the future. Insight was measured using the Birchwood Insight Scale (BIS), internalized stigma with the Internalized Stigma of Mental Illness Scale (ISMIS), quality of life using the S-QoL 18, and wellbeing with the Warwick-Edinburgh Mental WellBeing Scale (WEMWBS). We focused on subscale scores rather than total scores to better reflect their multidimensional structure. For the WEMWBS scale, the total score was used. For more details on the variables included, see Supplementary Methods.

### 2.4 Statistical analysis

Data were loaded into R environment (32) R version 4.2.3 was employed, and the *caret (v* 7.0.1*), multiROC (v* 1.1.1*), fastshap* (v 0.1.1), and *shapviz (v* 0.9.7*)* packages were utilized.

To evaluate model performance and avoid information leakage during hyperparameter optimization, we implemented a two-step validation strategy. First, the dataset was randomly partitioned into a training set (70%) and a hold-out test set (30%) . The number of observations in the test set was *N* = 408. To maintain the representative nature of our dataset, the sample was stratified into five primary strata, and sampling was conducted within these strata. This ensured that the training and testing sets preserved the underlying distribution of the cohort.

Second, to handle missing data, we used the *mice* (*v* 3.18.0*)* package in R, employing a multiple imputation approach. Based on the proportion of missing data (6.38%), we did not conduct a complete case analysis due to potential statistical power loss, selection bias, and because missingness was likely not Missing Completely at Random (MCAR) (33). Instead, we performed multiple imputations using chained equations. The imputation model used included predictive mean matching for continuous variables, logistic regression for binary variables, and polytomous regression for categorical variables. We generated 15 imputed datasets, with a maximum of 10 iterations per set to achieve convergence. All subsequent analyses, including machine learning and model explanation, were performed on the 15 imputed datasets, and the results were aggregated considering their variability (mean, standard deviation, and ranges). Imputation was performed within each dataset to preserve the independence between training and test sets.

Third, we used a ML algorithm to characterize multivariate patterns associated with recovery stages, using a random forest model (34). Random forests were preferred because they can model non-linear effects and interactions between predictors. Model training and hyperparameter tuning were conducted using 10-fold cross-validation within the training set. We employed the *caret* package to tune the following hyperparameters: the number of variables randomly sampled at each split (*mtry*), the minimum number of observations in terminal nodes (*min.node.size*), and the criterion for node splitting (*splitrule*). The number of trees was fixed at 500. We employed the adaptive sampling procedure proposed by Kuhn (35) for tuning hyperparameters in an efficient way, and subsequently evaluated the performance of the model in the training and test sets using Accuracy, Cohen's Kappa, and multiclass Area Under the Curve (mAUC). Multiclass AUC was computed by averaging pairwise one-vs.-one AUC values across outcome categories (see Supplementary Methods). Finally, SHAP (SHapley Additive exPlanations) values were used to characterize how sociodemographic and clinical variables contributed to stage classifications. SHAP is often described as a model-agnostic explanation approach with a solid theoretical foundation (36). SHAP estimates the contribution of each feature to model outputs by computing its marginal effects across all possible combinations of features, allowing examination of how features were associated with stage classifications. In this analysis, higher SHAP values for a given variable and observation indicate a stronger association of that variable with classification into a specific recovery stage. We aggregated absolute SHAP values at the population level to summarize the relative contribution of variables across stage classifications. Next, we generated univariate dependence plots for the 10 variables with the highest SHAP values, illustrating the relationship between raw feature values and their corresponding SHAP values.

## 3 Results

Supplementary Table 2 shows the sociodemographic and clinical characteristics of the 1,361 participants in the study. The mean age was 32.5 years (SD = 9.7) and 25.1% of the sample was female. Most of the participants were unemployed (93.1%), resided in their personal (47.3%) or family home (40.6%), and had a duration of illness of more than 10 years in 46.1% of the cases. 24.4% of patients had psychiatric comorbidity and 27.8% a history of suicide attempts. Regarding primary diagnosis, schizophrenia was the most frequent diagnosis, affecting 923 participants (67.8%).

The distribution of participants across the five STORI stages is presented in Supplementary Table 3. Overall, 60% of individuals were classified in the later stages of personal recovery (Rebuilding and Growth), reflecting the characteristics of a population engaged in PSR.

### 3.1 Performance of ML model

The random forest model showed high performance in internal validation. A mean accuracy (Accuracy) of 94.3% (SD = 2.8%), a kappa coefficient of 0.92 (SD = 3.8%), and an area under the ROC curve (AUC) of 0.997 (SD = 0.0015) were obtained on the training set, indicating an excellent fit to the training data (Table 1).

**Table 1:** Model performance for cross-validation on the training set and evaluation on a hold-out test set.

Dataset	Mean accuracy	SD accuracy	Mean kappa	SD kappa	Mean AUC	SD AUC	NIR
Internal (Train)	0.9435	0.0285	0.9242	0.0384	0.9971	0.0015	0.360
Hold-out test set (Test)	0.4984	0.0099	0.3033	0.0159	0.7242	0.0065	0.361

AUC, Area Under Curve; SD, Standard Deviation.

However, in the hold-out test set, model performance was more modest, with a mean accuracy of 49.8% (SD = 1.0%), a kappa of 0.30 (SD = 1.6%), and an AUC of 0.724 (SD = 0.0065). Given the five-category nature of the STORI outcome (chance-level accuracy = 20%), these results indicate that the model captured non-random structure in the data. Moreover, given the class imbalance, model accuracy was interpreted relative to the No Information Rate (NIR = 0.361), corresponding to a naive classifier predicting only the majority class. The observed test accuracy of 0.498 therefore exceeds this baseline, while remaining indicative of modest discriminative performance across five stages.

Class-specific performance metrics for the test set are presented in Supplementary Table 4. Sensitivity values ranged from 0.003 (SD = 0.008) for X3 to 0.826 (SD = 0.028) for X5. Balanced accuracy values ranged from 0.500 (SD = 0.004) to 0.770 (SD = 0.025) across classes. Precision (positive predictive value) ranged from 0.107 (SD = 0.197) to 0.588 (SD = 0.027), and F1 scores ranged from 0.042 (SD = 0.001) to 0.686 (SD = 0.014).

The column-wise percentage confusion matrix for the test set is shown in Supplementary Table 5. For class X1, 62.1% of cases were correctly classified; misclassification occurred mainly toward X2 (21.8%), followed by X4 (17.3%). For class X2, 17.9% of cases were correctly classified; misclassification occurred toward X1 (21.8%), X4 (36.4%), X3 (1.1%), and X5 (22.8%). For class X3, 0.3% of cases were correctly classified; most were misclassified as X4 (45.6%) and X5 (33.6%). For class X4, 36.2% of cases were correctly classified; misclassification occurred toward X5 (52.1%), X2 (4.7%), X1 (7.1%). For class X5, 82.6% of cases were correctly classified; misclassification occurred toward X4 (13.9%), X3 (0.0%), X2 (1.3%), and X1 (2.3%).

### 3.2 Contribution of each variable to each STORI stage

**Figure 1:**
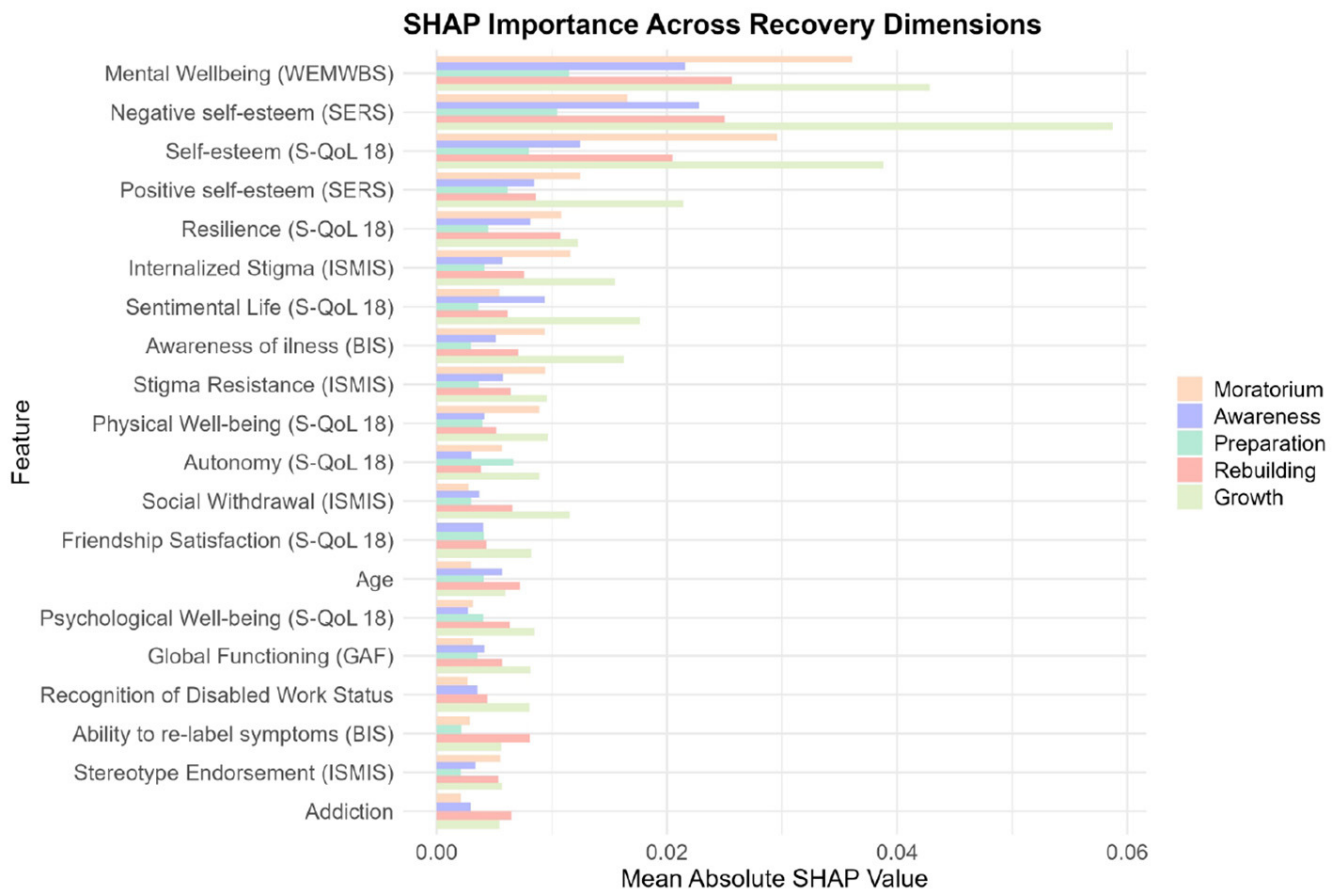
Multivariate characterization of STORI recovery stages based on variable importance. Self-esteem was assessed using three indicators: positive self-esteem (SERS), negative self-esteem (SERS), and self-esteem (S-QoL), the latter reflecting optimism and confidence in the future. BIS, Birchwood Insight Scale; GAF, Global Assessment of Functioning; ISMIS, Internalized Stigma of Mental Illness Scale; SERS, Self-Esteem Rating Scale; S-QoL 18, Quality of Life Short Version; WEMWBS, Warwick-Edinburgh Mental WellBeing Scale.

Interpretation of the models using SHAP values identified the variables involved in the characterization of STORI stage classifications. These variables included specific dimensions of self-esteem (positive, negative self-esteem with the SERS scale and optimism and confidence in the future with the S-QoL 18), resilience, and mental wellbeing. In contrast, sociodemographic and clinical variables showed comparatively limited involvement in stage-associated patterns across recovery profiles. A detailed representation of the contribution of each variable to each stage of recovery is presented in Figure 1. Complementarily, Table 2 provides a concise overview of the main stage-associated characteristics identified in the present sample.

**Table 2:** Stage-associated recovery profiles in the present sample, derived from multivariate patterns identified using SHAP.

STORI stage	Stage-associated recovery characteristics
Moratorium	High negative self-esteem, high internalized stigma, low psychological wellbeing, and positive self-esteem.
Awareness	Moderate psychological wellbeing, persistent negative self-esteem, with emerging positive self-esteem, and relational satisfaction.
Preparation	Less distinctive recovery-related pattern, with persistent contributions of mental wellbeing and self-esteem, and increasing relevance of autonomy.
Rebuilding	Intermediate-to-high psychological wellbeing, increased resilience, and more neutral self-esteem levels.
Growth	High psychological wellbeing, high positive self-esteem, low negative self-esteem, greater relational satisfaction.

#### 3.2.1 Moratorium

**Figure 2:**
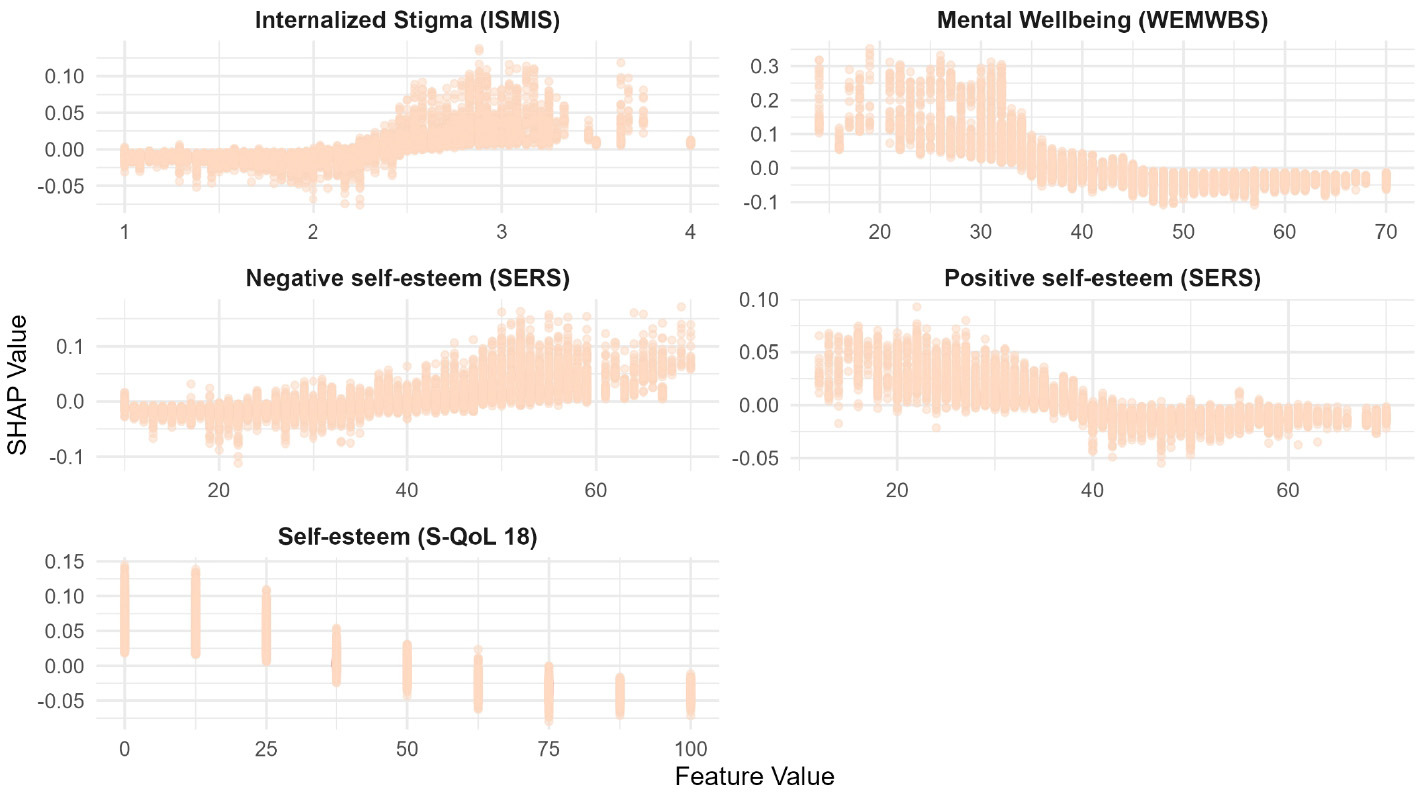
One-way SHAP dependence plots illustrating the five variables contributing most to the Moratorium stage classification. ISMIS, Internalized Stigma of Mental Illness Scale; SERS, Self-Esteem Rating Scale; S-QoL 18, Quality of Life Short Version; WEMWBS, Warwick-Edinburgh Mental WellBeing Scale. Self-esteem was assessed using three indicators: positive self-esteem (SERS), negative self-esteem (SERS), and self-esteem (S-QoL), the latter reflecting optimism and confidence in the future.

In the Moratorium stage, higher positive SHAP values were associated with high scores on negative self-esteem and internalized stigma. However, these associations were non-linear, with SHAP values increasing mainly at moderate to high levels of internalized stigma and negative self-esteem. In addition, lower positive self-esteem and lower wellbeing were associated with higher SHAP values (see Figure 2).

#### 3.2.2 Awareness

Supplementary Figure 1 shows the SHAP dependence plots for the Awareness stage. Mental wellbeing showed a prominent stage-associated pattern. Negative self-esteem also showed a clear positive association. In contrast, positive self-esteem and sentimental life (S-QoL-18 scale) were associated with higher SHAP values for this stage.

#### 3.2.3 Preparation

In the Preparation stage, SHAP values were generally of moderate magnitude, indicating a reduced overall discriminative separation, consistent with the low classification performance observed for this stage. Autonomy showed a clear stage-associated pattern in this classification. Mental wellbeing showed a distribution with slight positive variations at intermediate levels of the scale, as did negative self-esteem. Higher levels of positive self-esteem were associated with lower SHAP values (see Supplementary Figure 2).

#### 3.2.4 Rebuilding

Mental wellbeing and resilience exhibited non-linear patterns in SHAP values for the Rebuilding stage (Supplementary Figure 3). Specifically, SHAP values rose significantly once mental wellbeing and resilience reached intermediate levels.

#### 3.2.5 Growth

**Figure 3:**
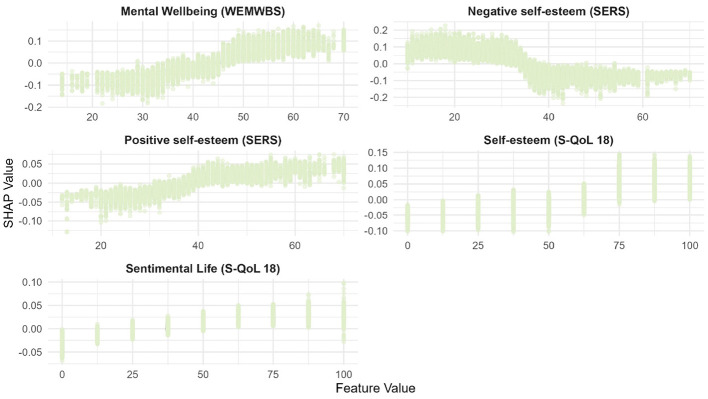
One-way SHAP dependence plots illustrating the five variables contributing most to the Growth stage classification. SERS, Self-Esteem Rating Scale; S-QoL 18, Quality of Life Short Version; WEMWBS, Warwick-Edinburgh Mental WellBeing Scale. Self-esteem was assessed using three indicators: positive self-esteem (SERS), negative self-esteem (SERS), and self-esteem (S-QoL), the latter reflecting optimism and confidence in the future.

A clear positive association was observed between mental wellbeing and SHAP values, especially at higher levels of the scale (Figure 3). Positive self-esteem and satisfaction with sentimental life also were associated with higher SHAP values for this stage, whereas high negative self-esteem showed negative SHAP values.

## 4 Discussion

This study used ML as an exploratory analytical approach to characterize multivariate patterns of association between sociodemographic, clinical, and psychological variables and personal recovery stages classifications in people with SSD, using the STORI scale. Moreover, while prior studies have used ML to predict treatment response, symptom trajectories, or relapse risk (37, 38), to our knowledge, this is the first to apply explainable ML techniques to explore these associations in relation to recovery stage classifications.

The model showed modest-to-moderate performance, consistent with the conceptual complexity of personal recovery, while offering interpretable outputs through SHAP values. In line with this, the substantial conceptual overlap between STORI stages may contribute to the observed performance, as it reflects the difficulty of discriminating between closely related recovery profiles. Rather than representing clearly separated and independent categories, the STORI stages may capture partially overlapping experiential patterns (18). This interpretation is further supported by the class-specific performance results and the confusion matrix, which showed that most misclassifications occurred between adjacent or conceptually related recovery stages. This limitation was particularly evident for the Preparation stage, which showed very low sensitivity and frequent misclassification into adjacent stages, consistent with overlapping recovery profiles. Furthermore, the marked difference between training and test performance indicates that the model should not be interpreted as a robust predictive classifier, but rather as an exploratory tool for identifying structured patterns of association.

Across all five STORI stages, psychological variables, particularly self-esteem, and psychological wellbeing, emerged as prominent contributors in the SHAP-based exploratory patterns. These findings are consistent with previous studies that have shown that positive psychological constructs such as self-esteem and wellbeing were key predictors of personal recovery in people with schizophrenia and are strongly associated with variation in STORI stage classifications (39–41). This finding should be interpreted in light of the theoretical framework of personal recovery, in which clinical recovery has been primarily linked to biochemical improvement and symptomatic reduction, functional recovery to the improvement of social and occupational functioning, and personal or subjective recovery to the strengthening of psychological resources, identity, and sense of purpose in life. From this perspective, an individual may show different levels of clinical, functional, and personal recovery domains at a given time point (42, 43).

In individuals classified in the Moratorium stage, the explanatory patterns highlighted self-stigma, low self-esteem, and reduced psychological wellbeing as central features. This stage, characterized by hopelessness and a loss of sense of personal identity, seems to be particularly influenced by the person's self-perception and negative beliefs about disease stereotypes. This could significantly hinder engagement with recovery-related processes (44). Conversely, positive self-esteem and psychological wellbeing could function as protective resources and facilitate openness to change. These findings highlight the importance of targeting self-stigma and self-esteem as central components of early recovery-oriented interventions (45, 46). A more intermediate profile, such as the Awareness stage, appeared to reflect a transition toward greater understanding and incipient participation in recovery, although with less clearly differentiated explanatory patterns. Regarding the Preparation stage, the model did not provide sufficiently reliable estimates, so the related findings should be interpreted with caution. In contrast, in individuals classified in the Rebuilding and Growth stages, the model also identified the contribution of factors such as social support and resilience. This pattern is consistent with previous studies that highlight these factors as predictors of personal recovery (47, 48). Notably, certain psychological dimensions, such as self-esteem and psychological wellbeing, contributed to multiple recovery stages, although the direction and relative prominence of their SHAP contributions varied across stages. This pattern is consistent with the conceptual overlap between STORI stages.

Interestingly, traditional clinical and sociodemographic factors such as age, gender, age at onset, number of psychiatric admissions, history of suicide attempts, or duration of illness contributed minimally to the model's predictions. This is in contrast with earlier research, which has often emphasized these characteristics as prognostic indicators of recovery or functional outcomes (49). For instance, while these variables may play a role in symptom remission or functioning (50), our results suggest that they are less informative when it comes to personal recovery. In line with this, Yanos et al. (44) found that, in the context of personal recovery, only small to moderate negative correlations existed with symptom severity and functional recovery. This interpretation is further supported by a recent meta-analysis by de Winter et al. (12), which showed that duration of illness does not significantly influence long-term changes in personal recovery. Taken together, these findings suggest that chronicity-related variables contribute relatively little to personal recovery outcomes. In contrast, social and functional variables (e.g., social support, employment, functioning) have been shown to be positively associated with personal recovery, although typically with smaller and less consistent effect sizes compared to psychological dimensions (25). In the present study, their relatively limited contribution may reflect the subjective and self-reported nature of personal recovery constructs, which are more closely aligned with psychological domains. This pattern is broadly consistent with recovery-oriented frameworks emphasizing subjective processes in personal recovery (51).

On the other hand, the interpretation of the limited contribution of sociodemographic factors should be contextualized within the specific characteristics of our sample, which was mostly composed of individuals engaged in psychosocial rehabilitation, with a high proportion of men and classified in advanced stages of recovery. However, previous research has suggested that these factors may influence recovery-related outcomes, although their effects remain inconsistent across studies (25, 52). These inconsistencies suggest that sociodemographic factors may exert differential influences on personal recovery depending on the stage of the illness and the timing of assessment. For instance, Owusu et al. (52) reported gender- and age-related differences in recovery-related outcomes in psychiatric inpatients assessed prior to discharge, highlighting the relevance of sociodemographic vulnerability during earlier or more unstable phases of care. Future studies should therefore examine personal recovery across different clinical stages and more heterogeneous samples to better clarify the context-dependent role of sociodemographic factors and improve generalizability.

The use of ML models allowed us to explore how the most relevant variables behaved across STORI stages classifications. Our aim was to use ML as a tool to reflect the dynamic and multidimensional nature of the recovery process through patterns of association between variables and stages. The contributions observed through SHAP suggest that recovery could be best understood as a configuration of co-occurring psychological and social processes rather than a sequential trajectory. These SHAP-based contributions should be interpreted as approximate explanatory patterns that reflect multivariate configurations of recovery-related dimensions. While it is true that the STORI emphasizes psychological aspects of recovery, our approach allows us to observe the dynamic contribution of different variables across stages. For example, although self-esteem showed a strong contribution to stage classification, its relative weight varied across stages, being less predominant in individuals classified in the Rebuilding and Growth stages, where resilience and social support showed greater relevance. Such nuanced insights cannot be obtained through simple correlations, reinforcing the novelty of our contribution. It is important to note that some of the variables with the greatest contribution overlap conceptually with the dimensions that define the STORI stages. But their influence should be understood as a reflection of conceptual coherence within the framework of personal recovery, rather than as evidence of a causal relationship or an independent predictive effect. In this context, the Rebuilding and Growth stages should not be interpreted as a simple “higher dose” of recovery or as a replacement of earlier stages. Rather, individuals may simultaneously endorse experiences characteristic of multiple stages (28), reinforcing the overlapping nature of personal recovery.

From a clinical perspective, the results suggest that individuals classified in different STORI stages present distinct recovery profiles defined by specific combinations of psychological characteristics. Profiles associated with higher levels of self-stigma and lower psychological wellbeing may call for increased clinical attention to internal psychological resources, whereas profiles marked by greater wellbeing, resilience, and social satisfaction may benefit from support that consolidates social participation and community integration. These stage-associated patterns may help inform clinical understanding of personal recovery, while avoiding assumptions about sequential trajectories or intervention effects.

This study has several limitations. First, the data come from a specific rehabilitation context in France, which may limit generalizability to other cultural or healthcare systems. The importance of contextual and cultural factors is highlighted by findings from a Polish study using the STORI scale (53), where none of the participants reached the Growth stage and only 11% were in the Rebuilding stage, in contrast to our sample and those from Australia and the UK (18, 26). Furthermore, cultural values may influence which factors are most salient in the recovery process. For example, Latin American cultures often emphasize collectivist values like solidarity, family support, and interdependence (54, 55), which may shape recovery experiences differently than in individualistic societies where most recovery frameworks were developed. Exploring these variables in future studies could enrich our understanding of the recovery process and inform more comprehensive intervention strategies. Second, the cross-sectional nature of the design prevents establishing causal relationships. Future studies adopting longitudinal designs could provide stronger insights into how predictors dynamically shape recovery trajectories. Third, the model showed more modest performance during the testing phase, with the gap between training and test performance suggesting overfitting (56). Therefore, the current results should be considered as exploratory. Future work could explore alternative algorithms, such as gradient boosting, to improve generalizability and robustness. Fourth, although multiple imputations were used to handle missing data, potential biases related to the imputation process cannot be ruled out. Fifth, while the model was able to capture a significant proportion of the variance, a considerable portion remains unexplained. This suggests that additional factors (such as environmental context, therapeutic relationships, trauma history, spirituality, or existential dimensions) may play a critical role in shaping the recovery trajectory (57). The absence of these constructs may limit the comprehensiveness of our understanding of the recovery process, as these factors have been shown to play a significant role in personal recovery. Furthermore, although specific measures of neurocognition were not included, existing evidence suggests that its association with personal recovery is limited and less consistent than its well-established links with clinical and functional outcomes. Some studies have not shown a clear association between neurocognition and personal recovery (25), while more recent work suggests that any influence may be indirect, for example, through functioning or participation, rather than a direct factor in subjective recovery (58). That said, the inclusion of neurocognitive measures in future studies could enrich the understanding of the phenomenon. In addition, in this study, affective symptoms were not directly assessed using a specific standardized measure. Some studies indicate that affective symptoms show a moderate negative association with personal recovery and may be more strongly related to subjective recovery than other symptom dimensions in psychosis (25, 52). A specific measure of affective/depressive symptoms validated for schizophrenia, such as the Calgary Depression Scale for Schizophrenia, could have improved the performance of our model. Sixth, the STORI is an instrument with documented conceptual and psychometric limitations (18). Although it has been widely used in both clinical and research settings, it has also received criticism regarding conceptual overlap between stages. This overlap may limit the discriminant separability of STORI stage classifications and contribute to reduced classification performance, particularly in independent test samples. Accordingly, the present findings should be interpreted as reflecting discrimination between partially overlapping recovery profiles, rather than the existence of fully distinct recovery categories. Finally, some constructs (such as resilience or autonomy) were measured using subscale scores from broader instruments like the S-QoL 18. This limited item coverage may affect the precision and validity of the associated predictors. This suggests that the inclusion of more specific instruments to assess these constructs (such as dedicated scales for resilience) could improve the quality of predictions in future models. A further limitation concerns the presence of correlated predictors, which can affect the stability and interpretability of SHAP values. According to Aas et al. (59), correlated predictors can significantly distort SHAP attributions, as most computationally viable SHAP frameworks implicitly assume predictor independence. This is especially relevant in our dataset, where several psychological variables are conceptually and statistically related. Therefore, the observed explanatory patterns should be understood as approximations, rather than precise estimates of the contributions of individual features.

## 5 Conclusion

This study demonstrates the feasibility of using ML models to explore the structure of personal recovery in people with SSD. The model captures patterns of association that reflect how different domains relate to recovery stages as operationalized by the STORI. The modest performance observed in the independent test set underscores that STORI stage classifications represent partially overlapping recovery profiles rather than clearly separable or predictive categories, reflecting conceptual and psychometric limitations of stage-based operationalizations of personal recovery. Across STORI stage classifications, the identified recovery profiles were mainly characterized by psychological self-report domains, consistent with the conceptual proximity between these constructs and the STORI operationalization of personal recovery. By using explainable ML techniques, this work provides an exploratory representation of how different domains contribute to recovery stage classifications. These findings are clinically relevant for characterizing stage-associated personal recovery profiles, highlighting differential configurations of psychological resources across STORI classifications. In particular, profiles marked by higher self-stigma and lower wellbeing are distinguished from those characterized by higher wellbeing, resilience, and greater satisfaction with relational and community life.

## Data Availability

The data analyzed in this study is subject to the following licenses/restrictions: AP-R and GB have full access to all data in the study and take responsibility for the integrity of the data and the accuracy of the analyses. The data that support the findings of this study are not publicly available due to privacy or ethical restrictions but are available from the corresponding author upon reasonable request. Requests to access these datasets should be directed to Guillaume Barbalat, guillaumebarbalat@gmail.com.
