# Supplementary material for: Mapping personal recovery in schizophrenia spectrum disorders: an exploratory machine learning study of self-reported stage classifications
Source: Front Public Health. 2026 Jun 15;14:1822191. doi: 10.3389/fpubh.2026.1822191 (PMC13310769; doi:10.3389/fpubh.2026.1822191)
Supplement: Supplementary file 1 [file Data_Sheet_1.pdf]

Supplemental Materials

Supplementary Methods. Description of recovery-related variables

Supplementary Methods. Model evaluation

Supplementary Table 1. Correlations between STORI recovery stages

Supplementary Table 2. Socio-demographic and clinical characteristics of participants

Supplementary Table 3. Distribution of participants across STORI recovery stages

Supplementary Table 4. Class-specific classification performance on the test set

Supplementary Table 5. Column-wise percentage confusion matrix for the test set across the 15 imputed datasets

Supplementary Figure 1. One-way SHAP dependence plots illustrating the five variables contributing most to the Awareness stage classification

Supplementary Figure 2. One-way SHAP dependence plots illustrating the five variables contributing most to the Preparation stage classification

Supplementary Figure 3. One-way SHAP dependence plots illustrating the five variables contributing most to the Rebuilding stage classification

References

Supplementary Methods. Description of recovery-related variables

Socio-demographic information included age, sex (male, female), and education level (no qualifications or basic vocational diploma, upper secondary school diploma, short cycle higher education diploma, master's degree or higher). Other socio-demographic characteristics included employment status (employed vs. unemployed); social marginalization (current, past, no); family situation (single, married/civil partnership/cohabiting, divorced/widowed); living situation (supervised apartment/home/other, family residence, personal residence, homeless/hospital/squat); parental status (no vs. yes); recognition as a disabled worker (no vs. yes); medico-legal history (no vs. yes).

Participants were recruited from 33 centers within a French psychosocial rehabilitation network. To ensure statistical robustness and reduce sparsity, centers with fewer than 50 observations were grouped into a single analytical category. This resulted in a total of 10 analytical units. Race/ethnicity, gender identity, and sexual orientation were not included in our set of predictors, as French regulations do not allow for the systematic collection of such sensitive data.

Clinical information included comorbid psychiatric diagnosis (no vs. yes); number of prescribed medications (1, 2, 3, 4, 5 or more); presence of an addiction (behavioral, substance-related addiction, both or none); origin of initial referral (public sector, patient, private sector, health and social care professional, other); number of psychiatric admissions (0, 1, 2, 3, 4, 5-10, 10 or more); duration of hospitalization (no hospitalization, 0-3 months, 3-6 months, 6-12 months, 12 months or more); number of suicide attempts (0,1,2,3,4 or more) ; duration of illness (less than 2 years, 2-5 years, 5-10 years, more than 10 years); functioning score based on the Global Assessment of Functioning (GAF) (1); severity of illness score based on the Clinical Global Impression scale (CGI) (2) and Medication Adherence Rating Scale (MARS) (3).

All hospitalizations referred exclusively to psychiatric admissions. The duration of hospitalization corresponded to the cumulative lifetime duration of psychiatric hospitalizations. Addiction diagnoses were established by experienced psychiatrists based on clinical interviews and medical records, in accordance with standard clinical practice within the network.

We also included information from self-reported scales and questionnaires.

Self-esteem was assessed using the Self-Esteem Rating Scale (SERS) (4), a 20-item self-report instrument rated on a 7-point Likert scale (1=never to 7=always). Scores for each dimension of self-esteem (positive and negative) were calculated by summing the responses to the corresponding items. Both subscales range from 10 to 70, with higher scores reflecting higher levels in the respective dimensions. Both positive and negative self-esteem were used as predictors. The SERS has been validated for use with French-speaking populations (5).

Insight was assessed using the Birchwood Insight Scale (BIS) (6), a self-rated instrument designed to measure insight in individuals with mental illness. The BIS includes 8 items rated on a 3-point Likert scale (0=disagree to 2=agree) and consists of items assessing three key dimensions: awareness of illness, recognition of the need for treatment, and ability to re-label symptoms. Instead of analyzing the total score, we focused on the subscale scores to capture these distinct dimensions. Higher scores represent greater insight and a score of 9 or more is considered good insight

The Internalized Stigma of Mental Illness Scale (ISMIS) (7) is a self-rated instrument designed to assess self-stigma from the perspective of individuals experiencing mental illness. It consists of 29 items rated on a four-point Likert scale (1=strongly disagree to 4=strongly agree) and is divided into five subscales: alienation, stereotype endorsement, perceived discrimination, social withdrawal, and stigma resistance. Higher scores indicate greater internalized stigma. Given its broad applicability across different health conditions, we specifically included subscale scores in our analysis.

Quality of life was measured with the S-QoL 18 (8), an 18-item self-administered questionnaire with a 5-point Likert scale. The S-QoL 18 consists of 18 items covering eight key dimensions: psychological well-being, self-esteem, autonomy, resilience, relationships with friends, relationships with family, romantic relationships, and physical well-being. Each dimension is scored from 0 to 100, with higher scores indicating a better quality of life.

Finally, the total score from the Warwick-Edinburgh Mental Well-Being Scale (WEMWBS) (9) was used. This is a 14-item scale developed to measure mental well-being. It uses a 5-point Likert scale, with total scores ranging from 14 to 70. The overall score is obtained by summing the scores of all items, with higher scores indicating greater levels of mental well-being.

Supplementary Methods. Hyperparameter tuning and Model evaluation.

Model training and hyperparameter tuning were conducted using 10-fold cross-validation within the training set, implemented via the caret package with the ranger random forest engine. We used caret's adaptive resampling procedure, which discards underperforming hyperparameter combinations early using a Bradley–Terry model, allowing computational resources to focus on the most promising configurations. The number of trees was fixed at 500. Model discrimination in the multiclass setting was assessed using the multiclass Area Under the Curve (mAUC). Because standard AUC is defined for binary outcomes, mAUC was computed using the method proposed by Hand and Till (2001) (10), which averages all pairwise one-versus-one AUCs between outcome categories.

Model performance was evaluated using Accuracy, Cohen's Kappa, and multiclass AUC. Accuracy reflects the proportion of correctly classified instances and is one of the oldest and most widely used metrics in classification research (11). Cohen's Kappa adjusts for the level of agreement that would be expected by chance, offering a more robust estimate of classification performance under class imbalance and is commonly applied when evaluating agreement between predicted and observed labels (12). Multiclass AUC was computed using the one-vs-rest strategy and subsequently macro-averaged across classes, following standard extensions of ROC-based metrics to multiclass classification, as implemented in widely used machine-learning frameworks such as scikit-learn (13). These indicators evaluate model performance for a multinomial outcome. Although the STORI was originally designed with an ordinal structure, we treated it as a multinomial (nominal) variable for this analysis; a detailed justification for this approach is provided in the main text.

Supplementary Table 1. Correlations between STORI recovery stages.

	**Moratorium**	**Awareness**	**Preparation**	**Rebuilding**	**Growth**
Moratorium	1	0.33	0.09	-0.24	-0.45
Awareness	0.33	1	0.8	0.44	0.18
Preparation	0.09	0.8	1	0.71	0.48
Rebuilding	-0.24	0.44	0.71	1	0.8
Growth	-0.45	0.18	0.48	0.8	1

Supplementary Table 2. Socio-demographic and clinical characteristics of participants

Variable	Mean (SD)	N (%)	Missing N (%)
**Age**	32.5 (9.7)	1361	0 (0.0)
**Sex**			0 (0.0)
Female		342 (25.1)	
Male		1019 (74.9)	
**Education level**			14 (1.0)
No qualifications or basic vocational diploma		615 (45.7)	
Upper secondary school diploma		413 (30.7)	
Short-cycle higher education diploma		238 (17.7)	
Master's degree or higher		81 (6.0)	
**Family situation**			44 (3.2)
Single		1086 (82.5)	
Married/civil partnership/cohabiting		169 (12.8)	
Divorced/widowed		62 (4.7)	
**Parental status**			44 (3.2)
No		1172 (89.0)	
Yes		145 (11.0)	

**Living situation**		47 (3.5)
Supervised appartement/home/other	112 (8.5)	
Family residence	534 (40.6)	
Personal residence	621 (47.3)	
Homeless/hospital/squat	47 (3.6)	
**Employment status**		53 (3.9)
Employed	90 (6.9)	
Unemployed	1218 (93.1)	
**Recognition as a disabled worker**		100 (7.3)
No	713 (56.5)	
Yes	548 (43.5)	
**Social marginalization**		57 (4.2)
Current	36 (2.8)	
Past	111 (8.5)	
No	1157 (88.5)	
**Medico-legal history**		61 (4.5)
No	1125 (86.5)	

Yes	175 (13.5)	
**Comorbid psychiatric diagnosis**		0 (0.0)
No	1029 (75.6)	
Yes	332 (24.4)	
**Diagnostic**		0 (0)
(F20.9) Schizophrenia	923 (67.8)	0 (0)
(F25) Schizoaffective disorder	252 (18.5)	0 (0)
(F23) Brief psychotic disorder	55 (4)	0 (0)
(F29) Unspecified psychotic disorder	39 (2.9)	0 (0)
(F20.81) Schizophreniform disorder	36 (2.6)	0 (0)
(F28) Other specified psychotic disorder	19 (1.4)	0 (0)
(F22) Delusional disorder	17 (1.2)	0 (0)
(F21) Schizotypal personality disorder	11 (0.8)	0 (0)
(F06) Substance-induced psychotic disorder	9 (0.7)	0 (0)
**Number of prescribed medications**		
No treatment	55 (4.0)	
1	399 (29.3)	

2	337 (24.8)	
3	275 (20.2)	
4	166 (12.2)	
5 or more	129 (9.5)	
**Addiction**		56 (4.1)
Behavioral	34 (2.6)	
Substance-related addiction	677 (51.9)	
Both	38 (2.9)	
None	556 (42.6)	
**Origin of initial referral (Addresser)**		49 (3.6)
Public sector	998 (76.1)	
Patient	56 (4.3)	
Private sector	191 (14.6)	
Health and social care professional	24 (1.8)	
Other	43 (3.3)	
**Number of psychiatric admission (s)**		128 (9.4)
0	93 (7.5)	
1	292 (23.7)	
2	248 (20.1)	

3	188 (15.2)	
4	114 (9.2)	
5-10 hospitalization	216 (17.5)	
10 or more	82 (6.7)	
**Duration of hospitalization**		232 (17.0)
No hospitalization	93 (8.2)	
0-3 months	336 (29.8)	
3-6 months	272 (24.1)	
6-12 months	216 (19.1)	
12 months or more	212 (18.8)	
**Duration of illness**		104 (7.6)
< 2 years	160 (12.7)	
2-5 years	232 (18.5)	
5-10 years	285 (22.7)	
10 or more years	580 (46.1)	
**Suicide attempts**		75 (5.5)

0		929 (72.2)	
1		201 (15.6)	
2		64 (5.0)	
3		41 (3.2)	
4 or more		51 (4.0)	
**MARS score total**	6.8 (2.0)		283 (20.8)
**GAF**	56.5 (13.6)		191 (14.0)
**Score CGI**	4.2 (1.1)		197 (14.5)
**SERS**			
SERS positive	40.1 (11.3)		186 (13.6)
SERS negative	35.4 (12.5)		183 (13.4)
**BIS**			
Awareness of mental illness	2.3 (1.3)		82 (6.0)
Ability to re-label symptoms	2.9 (1.1)		82 (6.0)
Recognition of a need treatment	3.3 (1.0)		85 (6.2)
**ISMIS**			
Stigma resistance	2.6 (0.6)		80 (5.8)
Alienation	2.2 (0.5)		88 (6.4)
Social Withdrawal	2.3 (0.7)		80 (5.8)

Perceived discrimination	2.2 (0.6)	82 (6.0)
Stereotype endorsement	1.8 (0.5)	81 (5.9)
**s-QoL 18**		
Self-Esteem	50.9 (28.3)	98 (7.2)
Resilience	59.2 (25.5)	102 (7.4)
Autonomy	61.5 (26.2)	101 (7.4)
Physical well-being	45.3 (27.0)	98 (7.2)
Family relationships	67.8 (27.6)	101 (7.4)
Relationship with friends	49.2 (29.6)	103 (7.5)
Sentimental life	35.7 (29.4)	109 (8.0)
Psychological well-being	53.4 (27.1)	99 (7.2)
**WEMWBS total**	43.6 (9.8)	71 (5.2)

Abbreviations: BIS=Birchwood Insight Scale; **GAF**= Global Assessment of Functioning; **CGI**= Clinical Global Impressions Scale **ISMIS=**Internalized Stigma of Mental Illness Scale; **SERS**=Self-Esteem Rating Scale; **S-QoL 18**= Quality of Life Short Version; **WEMWBS**= Warwick-Edinburgh Mental Well-Being Scale.

Supplementary Table 3. Distribution of participants across STORI recovery stages.

Recovery stage	N	%
Moratorium	187	13.7
Awareness	202	14.8
Preparation	151	11.1
Rebuilding	331	24.3
Growth	490	36

Supplementary Table 4. Class-specific classification performance on the test set.

Class	Sensitivity	Specificity	PPV	NPV	Precision	Recall	F1	Prevalence	Detection Rate	Detection Prevalence	Balanced Accuracy	AUC Class
X1	0.621 (0.059)	0.919 (0.012)	0.553 (0.023)	0.939 (0.008)	0.553 (0.023)	0.621 (0.059)	0.584 (0.026)	0.138 (0.000)	0.086 (0.008)	0.155 (0.018)	0.770 (0.025)	0.87 (0.006)
X2	0.179 (0.065)	0.951 (0.019)	0.387 (0.076)	0.870 (0.007)	0.387 (0.076)	0.179 (0.065)	0.238 (0.070)	0.147 (0.000)	0.026 (0.010)	0.068 (0.025)	0.565 (0.025)	0.75 (0.008)
X3	0.003 (0.008)	0.998 (0.003)	0.107 (0.197)	0.890 (0.001)	0.107 (0.197)	0.003 (0.008)	0.042 (0.001)	0.111 (0.000)	0.000 (0.001)	0.002 (0.003)	0.500 (0.004)	0.63 (0.015)
X4	0.362 (0.047)	0.765 (0.027)	0.330 (0.020)	0.789 (0.008)	0.330 (0.020)	0.362 (0.047)	0.344 (0.029)	0.243 (0.000)	0.088 (0.012)	0.266 (0.031)	0.563 (0.015)	0.66 (0.013)
X5	0.826 (0.028)	0.671 (0.045)	0.588 (0.027)	0.873 (0.013)	0.588 (0.027)	0.826 (0.028)	0.686 (0.014)	0.361 (0.000)	0.298 (0.010)	0.509 (0.037)	0.748 (0.014)	0.84 (0.005)

Note. Values are presented as *Mean* (*Standard Deviation*). PPV = positive predictive value; NPV = negative predictive value. F1 score represents the harmonic mean of precision and recall and reflects the balance between both metrics. AUC = area under the receiver operating characteristic curve. Classes correspond to recovery stages as defined by the Stages of Recovery Instrument (STORI): X1 = Moratorium, X2 = Awareness, X3 = Preparation, X4 = Rebuilding, and X5 = Growth.

Supplementary Table 5. Column-wise percentage confusion matrix for the test set across the 15 imputed datasets.

Prediction	X1	X2	X3	X4	X5
**X1**	62.1	21.8	10.8	7.1	2.3
**X2**	11.0	17.9	9.6	4.7	1.3
**X3**	0.2	1.1	0.3	0.0	0.0
**X4**	17.3	36.4	45.6	36.2	13.9
**X5**	9.4	22.8	33.6	52.1	82.6

Note. Values represent column-wise percentages averaged across the 15 imputed datasets. Columns correspond to observed classes and rows correspond to predicted classes. Classes correspond to recovery stages as defined by the Stages of Recovery Instrument (STORI): X1 = Moratorium, X2 = Awareness, X3 = Preparation, X4 = Rebuilding, and X5 = Growth.

Supplementary Figure 1. One-way SHAP dependence plots illustrating the five variables contributing most to the Awareness stage classification.

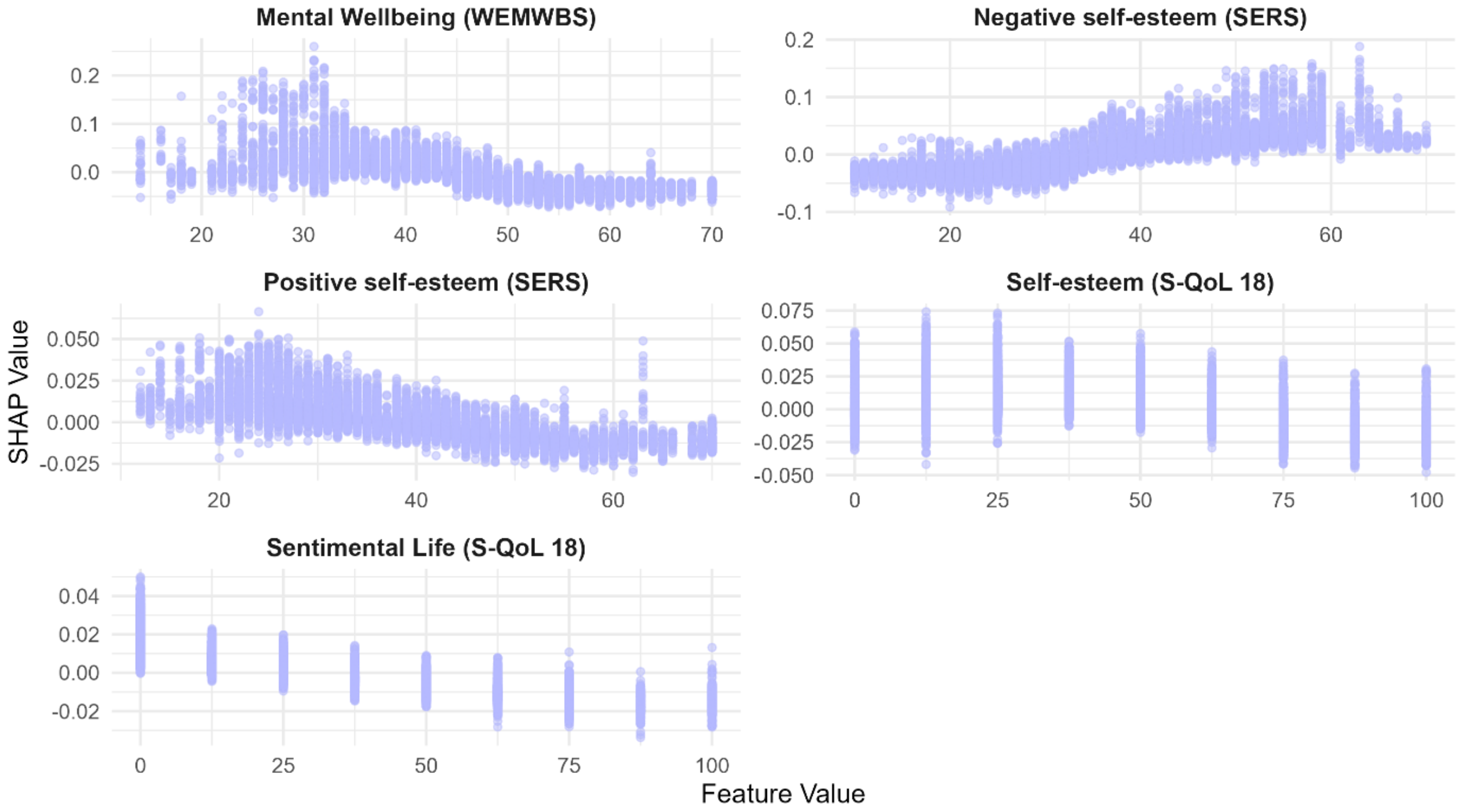

Abbreviations: SERS=Self-Esteem Rating Scale; **S-QoL 18**= Quality of Life Short Version; **WEMWBS**= Warwick-Edinburgh Mental Well-Being Scale.

Note. Self-esteem was assessed using three indicators: positive self-esteem (SERS), negative self-esteem (SERS), and self-esteem (S-QoL), the latter reflecting optimism and confidence in the future.

Supplementary Figure 2. One-way SHAP dependence plots illustrating the five variables contributing most to the Preparation stage classification.

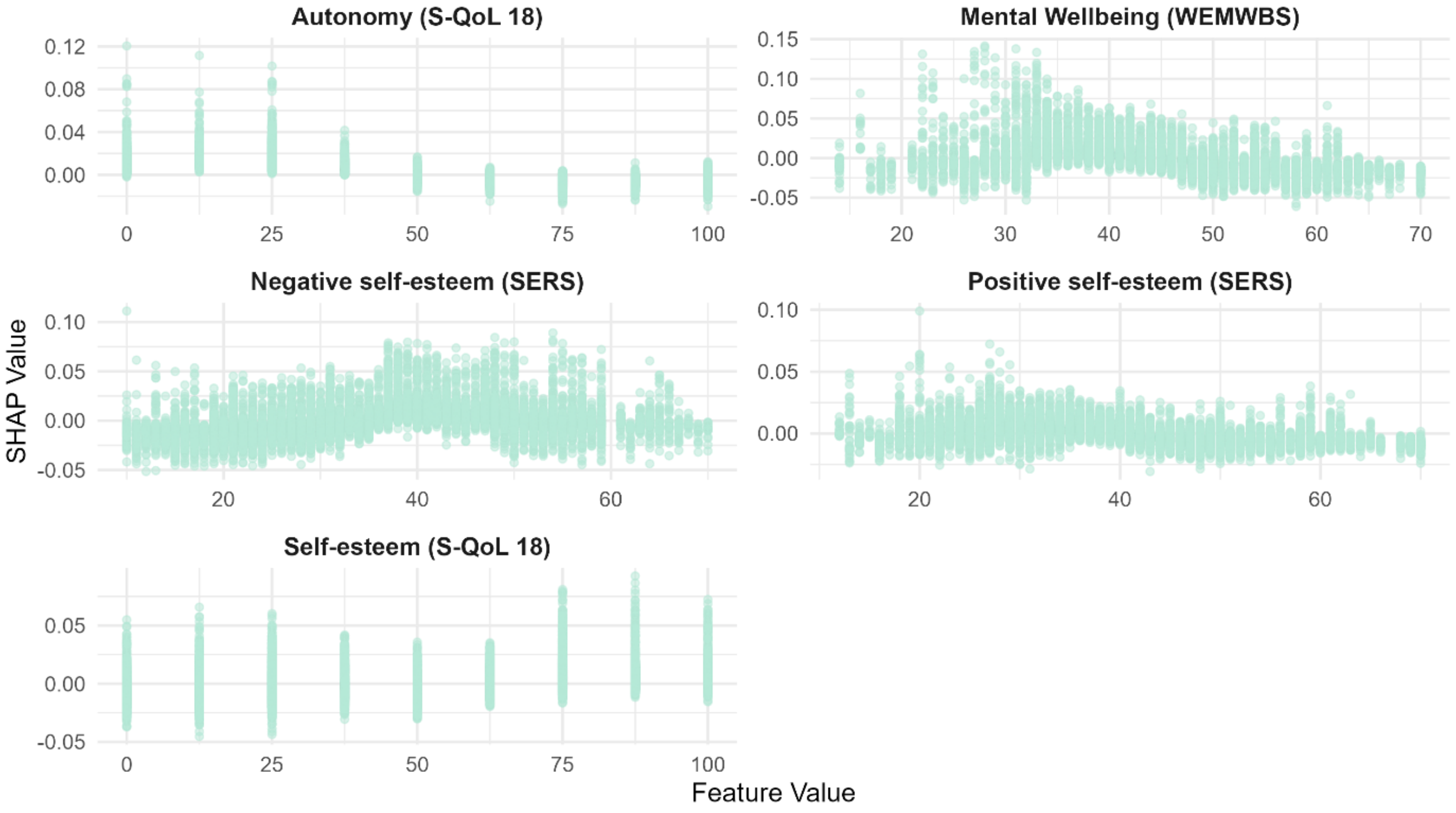

Abbreviations: SERS=Self-Esteem Rating Scale; **S-QoL 18**= Quality of Life Short Version; **WEMWBS**= Warwick-Edinburgh Mental Well-Being Scale.

Note. Self-esteem was assessed using three indicators: positive self-esteem (SERS), negative self-esteem (SERS), and self-esteem (S-QoL), the latter reflecting optimism and confidence in the future.

Supplementary Figure 3. One-way SHAP dependence plots illustrating the five variables contributing most to the Rebuilding stage classification.

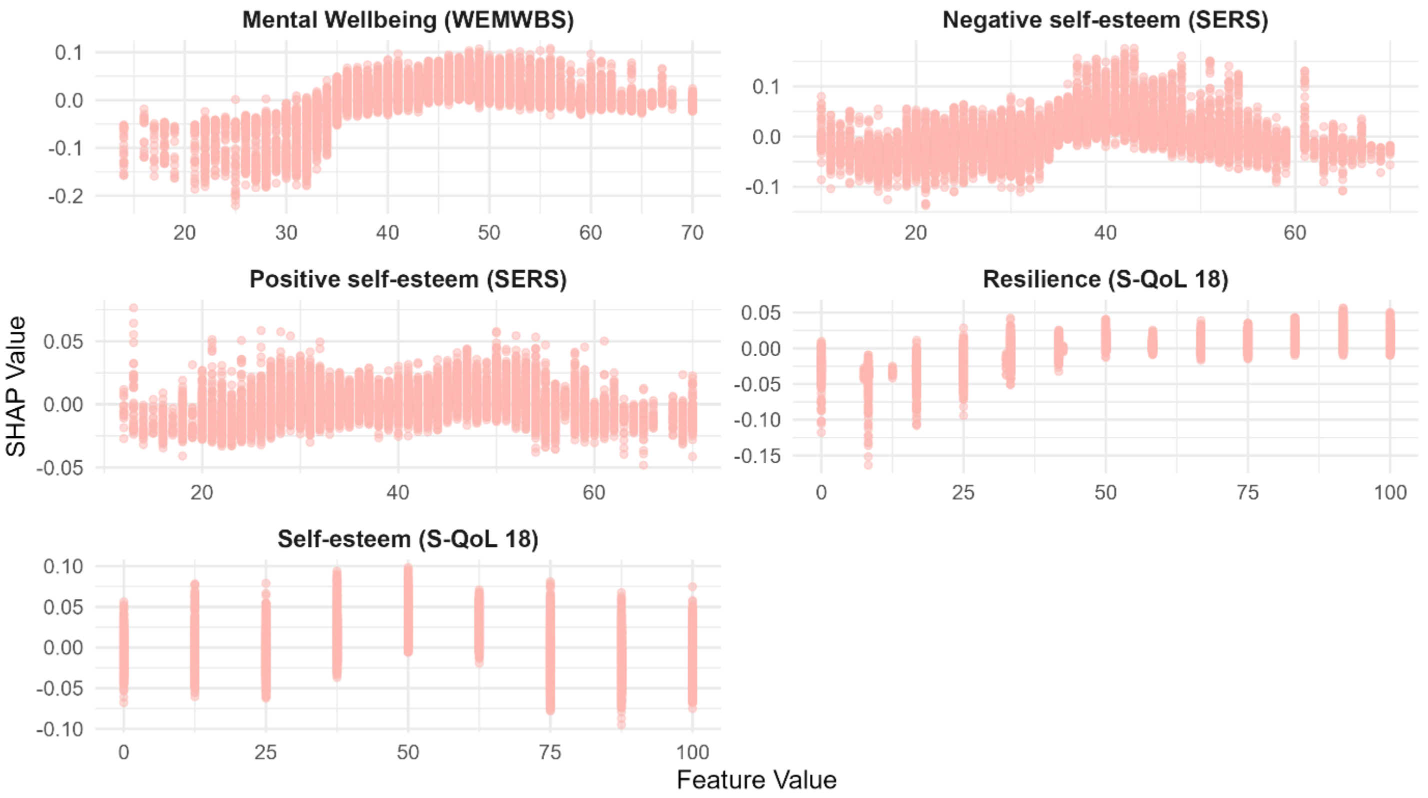

Abbreviations: SERS=Self-Esteem Rating Scale; **S-QoL 18**= Quality of Life Short Version; **WEMWBS**= Warwick-Edinburgh Mental Well-Being Scale.

Note. Self-esteem was assessed using three indicators: positive self-esteem (SERS), negative self-esteem (SERS), and self-esteem (S-QoL), the latter reflecting optimism and confidence in the future.

References

1. Endicott J, Spitzer RL, Fleiss JL, Cohen J. The Global Assessment Scale: A Procedure for Measuring Overall Severity of Psychiatric Disturbance. Archives of General Psychiatry. 1976 Jun 1;33(6):766–71. doi:10.1001/archpsyc.1976.01770060086012

2 Guy W. ECDEU assessment manual for psychopharmacology. US DEPARTMENT OF HEALTH, EDUCATION, AND WELFARE • Public Health Service Alcohol, Drug Abuse, and Mental Health Administration. 1976 Jan;3(2):307–8.

3. Thompson K, Kulkarni J, Sergejew AA. Reliability and validity of a new Medication Adherence Rating Scale (MARS) for the psychoses. 2000;(42).

4. Nugent WR, Thomas JW. Validation of a clinical measure of self-esteem. Vol. 3. 1993;3(2):191–207.

5. Lecomte T, Corbière M, Laisné F. Investigating self-esteem in individuals with schizophrenia: Relevance of the Self-Esteem Rating Scale-Short Form. Psychiatry Research. 2006 Jun 30;143(1):99–108. doi:10.1016/j.psychres.2005.08.019

6. Birchwood M, Smith J, Healy J, Macmillan F, Slade M. A self-report Insight Scale for psychosis: reliability, validity and sensitivity to change. Acta Psychiatrica Scandinavica [Internet]. 1994 [cited 2026 Mar 2]. Available from: https://onlinelibrary.wiley.com/doi/abs/10.1111/j.1600-0447.1994.tb01487.x?sid=nlm%3Apubmed

7. Boyd Ritsher J, Otilingam PG, Grajales M. Internalized stigma of mental illness: psychometric properties of a new measure. Psychiatry Research. 2003 Nov 1;121(1):31–49. doi:10.1016/j.psychres.2003.08.008

8. Boyer L, Simeoni MC, Loundou A, D'Amato T, Reine G, Lancon C, Auquier P. The development of the S-QoL 18: a shortened quality of life questionnaire for patients with schizophrenia. Schizophr Res. 2010 Aug;121(1-3):241-50. doi: 10.1016/j.schres.2010.05.019. Epub 2010 Jun 12. PMID: 20541912.

9. Tennant R, Hiller L, Fishwick R, Platt S, Joseph S, Weich S, et al. The Warwick-Edinburgh Mental Well-being Scale (WEMWBS): development and UK validation. Health and Quality of Life Outcomes. 2007 Nov 27;5(1):63. doi:10.1186/1477-7525-5-63

10. Hand DJ, Till RJ. A simple generalisation of the area under the ROC curve for multiple class classification problems. Machine Learning. 2001;45(2):171–186. doi:10.1023/A:1010920819831

11. ssin122. Understanding Diagnostic Metrics for Classifiers: Accuracy vs Cohen's Kappa vs ROC vs Precision and Recall vs F1 – Research Strategies and Skills [Internet]. 2024 Sep 20 [cited 2026 Mar 2]. Available from: https://my-hdr-blogs.shaveensingh.com/article/understanding-diagnostic-metrics-for-classifiers-accuracy-vs-cohens-kappa/

12. Widmann M. Cohen's Kappa: What It Is, When to Use It, and How to Avoid Its Pitfalls. The New Stack [Internet]. 2020 Aug 4 [cited 2026 Mar 2]. Available from: https://thenewstack.io/cohens-kappa-what-it-is-when-to-use-it-and-how-to-avoid-its-pitfalls/

13. Scikit-learn Developers. scikit-learn [Internet]. [cited 2026 Mar 2]. 3.4. Metrics and scoring: quantifying the quality of predictions. Available from: https://scikit-learn/stable/modules/model_evaluation.html
